# Investigating the relationship between Aerosol Optical Depth and Precipitation over Southeast Asia with Relative Humidity as an influencing factor

**DOI:** 10.1038/s41598-017-10858-1

**Published:** 2017-10-17

**Authors:** Daniel Hui Loong Ng, Ruimin Li, Srivatsan V. Raghavan, Shie-Yui Liong

**Affiliations:** 1 Department of Civil and Environmental Engineering, National University of Singapore, Singapore, Singapore; 2 Tropical Marine Science Institute, National University of Singapore, Singapore, Singapore; 3 Center for Environmental Sensing and Modeling, Singapore-MIT Alliance for Research and Technology, Singapore, Singapore; 4 Willis Re Inc., London, UK

## Abstract

Atmospheric aerosols influence precipitation by changing the earth’s energy budget and cloud properties. A number of studies have reported correlations between aerosol properties and precipitation data. Despite previous research, it is still hard to quantify the overall effects that aerosols have on precipitation as multiple influencing factors such as relative humidity (RH) can distort the observed relationship between aerosols and precipitation. Thus, in this study, both satellite-retrieved and reanalysis data were used to investigate the relationship between aerosols and precipitation in the Southeast Asia region from 2001 to 2015, with RH considered as a possible influencing factor. Different analyses in the study indicate that a positive correlation was present between Aerosol Optical Depth (AOD) and precipitation over northern Southeast Asia region during the autumn and the winter seasons, while a negative correlation was identified over the Maritime Continent during the autumn season. Subsequently, a partial correlation analysis revealed that while RH influences the long-term negative correlations between AOD and precipitation, it did not significantly affect the positive correlations seen in the winter season. The result of this study provides additional evidence with respect to the critical role of RH as an influencing factor in AOD-precipitation relationship over Southeast Asia.

## Introduction

There has been overwhelming evidence that humans are the dominant cause of climate change^1–3^. While most of the global warming phenomenon has been attributed to increasing emissions of greenhouse gases^4^, recent research has also shown that anthropogenic aerosols can impact the climate through changes in the global radiation and energy budget^5–7^. Besides the effects on earth’s energy budget, aerosols influence the climate by perturbing the hydrological cycle^8,9^. The complexities in the hydrological cycle and the lack of adequate observations make the effects of anthropogenic aerosols on the hydrological cycle little known compared to their effects on the radiation budget. Therefore, there has been an increasing interest in studying the interactions between anthropogenic aerosols and the most important element in the hydrological cycle, precipitation.

Atmospheric aerosols influence precipitation through the direct, indirect and semi-direct effects^10–12^. The direct effect refers to the scattering and absorption of incoming solar radiation by atmospheric aerosols. This effect reduces the amount of radiation which is able to reach the ground surface, thus, cooling the surface and affecting the atmospheric stability^11–14^ As such, evaporation processes and circulation patterns may also be affected and this leads to changes in precipitation.

The indirect effect is a mechanism by which aerosols modify the microphysical properties of cloud and hence, the radiative properties. There are two different indirect effects. The first indirect effect refers to the reduction in the size of the cloud condensation nuclei (CCN) due to the increase in the number of aerosols when there is a constant liquid water content^15^. These smaller cloud droplets result in a suppression of precipitation in shallow and short-lived clouds. The second indirect effect is an extension of the first indirect effect where the suppression of precipitation results in an increase in cloud lifetime. This lifetime extension of clouds in convective systems makes conditions favourable for extreme precipitation^16^.

The semi-direct effect is depicted as a mechanism by which absorbing aerosols heat the cloud in which they mix. Subsequently, the cloud droplets evaporate^17^ and this reduces cloud coverage. As a result, more solar radiation will reach the atmospheric layer under the clouds^18^ and increase the temperature in the lower atmosphere. This induces an increase in atmospheric stability under the aerosol plume, which may attenuate surface evaporation and the convection processes. Thus, there may be a decrease in rainfall. Due to these factors, there is still high uncertainty on the overall effects aerosols can have on precipitation.

A number of previous studies have investigated the influence of aerosols on precipitation. These studies have reported either positive or negative correlations between aerosol proxies (such as Aerosol Optical Depth, hereinafter referred to as AOD) and precipitation intensity^19,20^ based on observational data products. However, it is important to note that the observed correlation does not imply causality and that it could be ascribed to external factors. Such factors include wet deposition of aerosols due to precipitation^21,22^ and hygroscopic growth of aerosols due to condensation of water vapour on the aerosols with increasing relative humidity (RH)^23,24^. Numerous studies have reported that RH may influence the relationship between aerosols and precipitation^25,26^. Recent evidence also suggests that RH is one of the important drivers of precipitation-AOD relationship based on satellite-retrieved products^24,27^. RH represents the amount of water vapour in the air expressed as a percentage of the amount needed for saturation, at the constant temperature. Thus, it is an important measure of atmosphere saturation^28^ and is closely related to the microphysical processes in cloud development and in precipitation^29^. Therefore, it is appropriate to evaluate if RH is an influencing factor for the relationship between aerosols and precipitation.

Southeast Asia is known to have large temporal and spatial variances in AOD^30^, and also hosts one of the most complex cloud-precipitation systems in the world with convoluted meteorological scales and sharp geographic features^31^. Besides, Southeast Asia also has a highly dynamic hydrological system as precipitation is largely influenced by distinct monsoonal seasons and the shift in the Inter Tropical Convergence Zone^32^. Furthermore, Southeast Asia also has frequent widespread biomass burning events^33,34^ which emit huge amounts of aerosols into the atmosphere. These aerosols are then transported over the region causing transboundary smoke haze. Thus, it is important to understand if these aerosols have an impact on precipitation over this region.

There has been little discussion about the influence of RH on the relationship between aerosols and precipitation over Southeast Asia despite the region having distinctively high atmospheric humidity^35^ and convoluted aerosol and rainfall systems. Therefore, being one of the many studies that deal with the understanding of the relationship between aerosols and precipitation over Southeast Asia, this study delves into validating the influence of RH on the relationship between aerosols and precipitation on daily and monthly scales. This paper analyses 15 years (2001–2015) of satellite and gauge based rainfall products against AOD and RH over Southeast Asia. It is hoped that the main findings from this study would improve our understanding of causality between aerosols and precipitation in Southeast Asia.

## Methodology

### Study region

**Figure 1:**
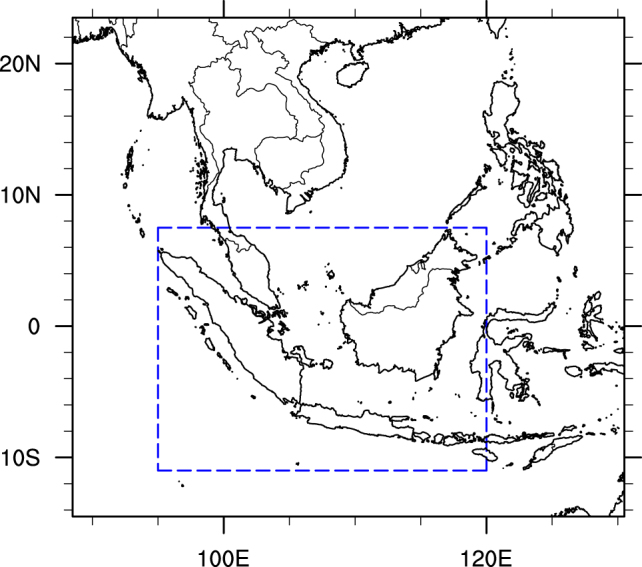
Southeast Asia domain used in this study. The dashed boundary denotes the Maritime Continent region. The map was generated using the NCAR Command Language (Version 6.3.0) [Software]. (2015). Boulder, Colorado: UCAR/NCAR/CISL/TDD. 10.5065/D6WD3XH5.

The Southeast Asian region (Fig. 1) in this study is bounded by the longitudinal coordinates of 88.5°E to 130.5°E and by the latitudinal coordinates of 23.5°N to 14.5°S. The domain includes both the Maritime Continent (Indonesia, Malaysia, Singapore, and Brunei) and the Continental Southeast Asia (Myanmar, Thailand, Cambodia, Laos, and Vietnam), which have been previously reported as the main contributors to the variance in atmospheric aerosol loading within Southeast Asia^30^ due to frequent biomass burning events.

### Data

The different observational data used in this study are described in this section. The AOD data were obtained from the Moderate Resolution Imaging Spectroradiometer (MODIS) instrument on-board the Terra satellite^36,37^. Precipitation data from both the Tropical Rainfall Measuring Mission (TRMM), which is a merged product from different satellites, and Climate Hazards Group InfraRed Precipitation with Station data (CHIRPS), which is a rain-gauge based product, were used. These precipitation data sets were chosen as previous studies that evaluated various precipitation products have ranked TRMM and CHIRPS among the best available gridded satellite products^38^. RH data from the ERA-Interim reanalysis product^39^ were also used in the study.

The datasets generated and/or analysed during the current study are available from the corresponding author on reasonable request.

### MODIS AOD

The data used in the analysis were the Level 3 global AOD data from MODIS and the data are available for the time period starting from February, 2000. These data have been used in numerous previous studies on aerosols^19,40,41^. In this study, data were obtained for the years from 2001 to 2015. The data have a spatial resolution of 1° × 1° at a daily temporal resolution. The daily data were also aggregated to obtain monthly data. Based on the recommendations of MODIS (https://modis-atmos.gsfc.nasa.gov/products/aerosol/format-content), the negative AOD data was kept for the monthly-scale analysis while only AOD data that had values larger than 0 were used for the daily-scale analysis^42^.

### TRMM precipitation

The data used in this study are the TRMM 3B42v7, based on the TRMM Multi-satellites Precipitation Analysis (TMPA) products^43^. TRMM data is available from 1998 to 2016 and data from 2001 to 2015 were used. These data are available on a 0.25° spatial grid between the coordinates 50°S to 50°N and 0° to 360°E. The TRMM daily data were downloaded from the data portal (accessible at https://pmm.nasa.gov/data-access/downloads/trmm) and monthly data were obtained by accumulating the daily values. Due to their 0.25° × 0.25° resolution, they were re-gridded using bi-linear interpolation into a spatial resolution of 1° × 1°, to match the AOD data for the partial correlation analysis.

### CHIRPS precipitation

CHIRPS was developed by the Climate Hazards Group at the University of California, Santa Barbara^44^ and it utilizes a few data inputs including that of TRMM. The data are available from 1981 to present and data from 2001 to 2015 were used. The data have a 0.25° × 0.25° spatial resolution at a daily temporal resolution. The daily precipitation data from CHIRPS were also re-gridded from 0.25° × 0.25° to 1° × 1° to be compared against the AOD data.

### ERA-Interim RH

Daily and monthly RH data sets, between the pressure levels of 700 mb and 850 mb, were obtained from the ERA-Interim reanalyses. These pressure levels were chosen as they cover the cloud formation heights in the Southeast Asia region^45^. This product is available from 1979 to present and the data from 2001 to 2015 were used in this study. These data were downloaded at a desired spatial resolution of 1° × 1° from the data portal (accessible at http://apps.ecmwf.int/datasets/data/) and were re-gridded to have the same grids as AOD and precipitation. The RH data were also averaged throughout the various pressure levels between 700 mb and 850 mb.

## Data Analysis

### Empirical Orthogonal Function analysis

The Empirical Orthogonal Function (EOF) analysis was used in order to examine and understand the spatial and temporal variability of AOD fields. EOF analysis is a type of eigenvector analysis which decomposes a signal or dataset in terms of orthogonal basis functions to obtain temporal variation patterns (Principal components, PCs) and spatial variation patterns (EOFs) separately. This method generates several uncorrelated temporal-spatial modes which usually are ordered in terms of their representations of data variance. As the EOFs and PCs are orthogonal within each mode^46^, this analysis allows the temporal and spatial variations of each mode to be analyzed separately. This eases the identification of the temporal and spatial properties^47^.

In a previous study over Southeast Asia, this EOF analysis has revealed that precipitation in this region features a strong monsoon signal^48–50^. As monsoon activities have been previously linked to the wet deposition processes that influence atmospheric aerosol concentration^51^, the current study would only focus on the effects of aerosols on precipitation. Therefore, EOF analysis was conducted only on the AOD dataset.

The complexity in the aerosol system in this region is mainly attributable to the heterogeneous emissions influenced by human activities and the perturbations caused by convoluted meteorological systems. Decomposing the spatial-temporal signals manifested in the AOD field by the EOF analysis for the period 2001–2015 may reveal the dominant patterns of the regional aerosol system. This will improve the understanding of the effects of aerosols on precipitation.

### Correlation analyses

Different types of correlation analysis were also applied to identify the relationships between AOD and precipitation. Correlation analysis is usually used to investigate the relationship between precipitation and AOD by examining the strength of a statistical correlation between the two variables^20,52,53^. The P-value derived from the t-test is also commonly used to estimate the statistical significance of the resulting correlations. In this study, the correlations were considered significant when the P-value was less than the significance level of 0.05.

In this study, a Pearson correlation analysis was first used to identify the relationship between the two leading PCs of AOD and precipitation on a monthly scale. Additionally, the correlation analysis was also repeated for the daily CHIRPS precipitation and AOD data to understand the relationship between aerosols and precipitation at a higher temporal resolution. Furthermore, the AOD and precipitation were categorized by seasons (December, January and February representing the winter season, March, April and May, the spring, June, July and August, the summer and September, October and November, representing the autumn season), before the correlation analyses were conducted for each season. Pearson correlation analysis was also conducted for AOD and RH in both the monthly and daily scale.

Additionally, partial correlation analysis was employed to detect possible influences of RH on the relationship between AOD and precipitation. Partial correlation involves calculating the correlation between two variables holding constant the external influences of a third^54^. In this study, the residuals approach for the partial correlation was employed. Residuals refer to the deviation (d_i_) between the actual magnitudes and the perfect correlation line for two variables. Therefore, the product moment correlation between the residuals of AOD and precipitation would show the relationship between AOD and precipitation independent of the influences of RH.

## Results and Discussions

### EOF Analysis

**Figure 2:**
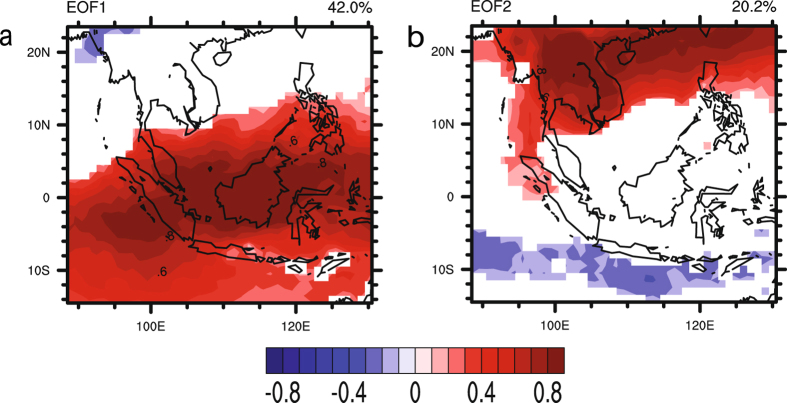
The EOFs of the two leading modes of EOF analysis on monthly AOD for 2001–2015: (**a**) EOF1; (**b**) EOF2. They are plotted as homogeneous correlation maps with correlation coefficient between AOD and its PCs plotted in each grid. Red indicates significant positive correlation (P-value < 0.05) while blue indicates significant negative correlation (P-value < 0.05). White denotes insignificant correlation (P-value > 0.05). These maps were generated using the NCAR Command Language (Version 6.3.0) [Software]. (2015). Boulder, Colorado: UCAR/NCAR/CISL/TDD. 10.5065/D6WD3XH5.

**Figure 3:**
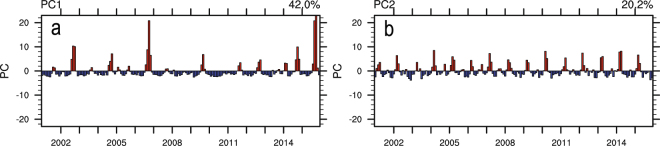
The PCs of the two leading modes of EOF analysis on monthly AOD for 2001–2015: (**a**) PC1 and (**b**) PC2. Red indicates positive phase of PCs while blue indicates negative phase of PCs. These plots were generated using the NCAR Command Language (Version 6.3.0) [Software]. (2015). Boulder, Colorado: UCAR/NCAR/CISL/TDD. 10.5065/D6WD3XH5.

Figure 2 presents the two leading modes of EOF analysis on AOD. Figure 3 shows the corresponding temporal variations of these modes, represented by the expansion coefficients, PC1 and PC2. These two leading EOF modes account for 62.2% of the total monthly AOD variance. The spatial patterns associated with these two AOD modes are shown (Fig. 2) as homogeneous correlation maps EOF1 and EOF2. EOF1 (Fig. 2a) exhibits a mono-pole pattern extending over the entire domain. As the EOFs shown in the Fig. 2 are represented by the correlation between AOD field and its two leading PCs, the square value of the correlations represents the variance of the AOD field explained by the two leading EOF modes. This mode accounts for up to 64% of the variance in the region of largest amplitude, namely, around the Maritime Continent. The expansion coefficients PC1 associated with this pattern is dominated by inter-annual fluctuations. The seasonal cycle shown in PC1 corresponds to the seasonal cycle of biomass burning over Maritime Continent^31^. Significant temporal variations which occurred in the summer half-year of 2002, 2006, and 2015 correspond to the strong warm phase in El Niño-Southern Oscillation (ENSO) periods. The intensity of the warm phase could also be observed through the Oceanic Niño Index (ONI) provided by National Oceanic and Atmospheric Administration (NOAA) of the United States of America (http://www.cpc.ncep.noaa.gov/products/analysis_monitoring/ensostuff/ensoyears.shtml). It is noted that the ONIs in the summer portion of the three years are higher than the ones in other periods. The strong warm phase is characterized by high pressure and dry conditions over the Maritime Continent. Positive values of the EOF1 indicate the spatial variation in phase with the variations of the corresponding temporal coefficient PC1. This variability in AOD matches the observed progress of fire activity throughout the Maritime Continent^31^ during fire-prone seasons. It can thus be suggested that the dry condition induced by ENSO is favorable for fire burning and hence, results in an increase in AOD over the Maritime Continent.

EOF2 (Fig. 2b) displays the other center of action for AOD variance around Continental Southeast Asia. The associated expansion coefficient time series PC2 consists of annual and inter-annual oscillations. Similarly, the positive values of the EOF2 indicate the spatial variation in phase with the variations of the PC2 while negative values indicate an out of phase variation. The possible explanation for the variability of AOD dominated by these patterns might be due to the seasonal cycle of biomass burnings in the Continental Southeast Asia. Biomass burning begins to develop in Cambodia in January-February and progresses through Thailand, Laos, and Myanmar, peaking in March, April and May, respectively^31,55,56^.

The two centers of action derived in the current study are consistent with those of Cohen^30^ where 12 years of monthly AOD data were used to investigate the aerosol climatology over Southeast Asia. He also validated the resulting EOF modes with observation data from the AErosol RObotic NETwork (AERONET) and concluded that there were two distinct biomass burning regions within Southeast Asia. When both EOF1 and EOF2 from this study were compared to the regions reported by Cohen, it was clear that these two leading modes of AOD were mainly induced by biomass burning activities over Southeast Asia.

Therefore, the following correlation analysis on the two PCs and monthly precipitation mainly reveals the relationship between precipitation and those aerosols emitted by biomass burning.

### Pearson Correlation Analysis between AOD and Precipitation

**Figure 4:**
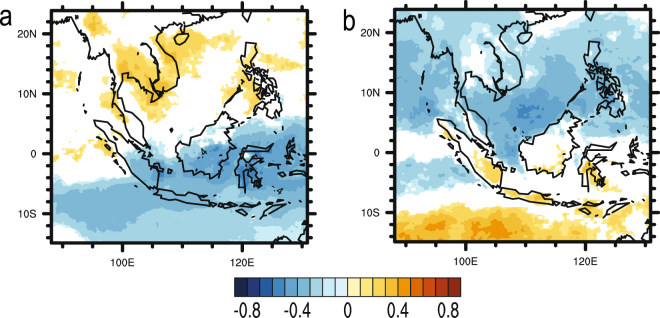
Correlation maps between TRMM monthly precipitation and the PCs of AOD for 2001–2015: (**a**) correlation between monthly precipitation and PC1; (**b**) correlation between monthly precipitation and PC2. Yellow indicates significant positive correlation (P-value < 0.05) while blue indicates significant negative correlation (P-value < 0.05). White denotes insignificant correlation (P-value > 0.05). These maps were generated using the NCAR Command Language (Version 6.3.0) [Software]. (2015). Boulder, Colorado: UCAR/NCAR/CISL/TDD. 10.5065/D6WD3XH5.

Figure 4 presents the correlation coefficients between monthly TRMM precipitation and the PCs of AOD at each grid point over Southeast Asia. In the left panel (Fig. 4a), the precipitation is strongly correlated with the PC1 over the Maritime Continent; while the right panel (Fig. 4b) exhibits a strong correlation between precipitation and PC2 over Continental Southeast Asia. In Fig. 4a, a significant correlation of up to −0.59 is observed over the region while a significant correlation of up to −0.54 is observed in Fig. 4b. The negative correlation coefficients indicate that the spatial variation of precipitation field is out of phase with the variations of the PCs of AOD. Given the EOFs of AOD field shown in Fig. 2, it is noted that the variability of precipitation is negatively correlated with the biomass-burning induced variability of AOD over the Maritime Continent as well as the Continental Southeast Asia. These results suggest that changes in precipitation were likely due to aerosol interactions.

**Figure 5:**
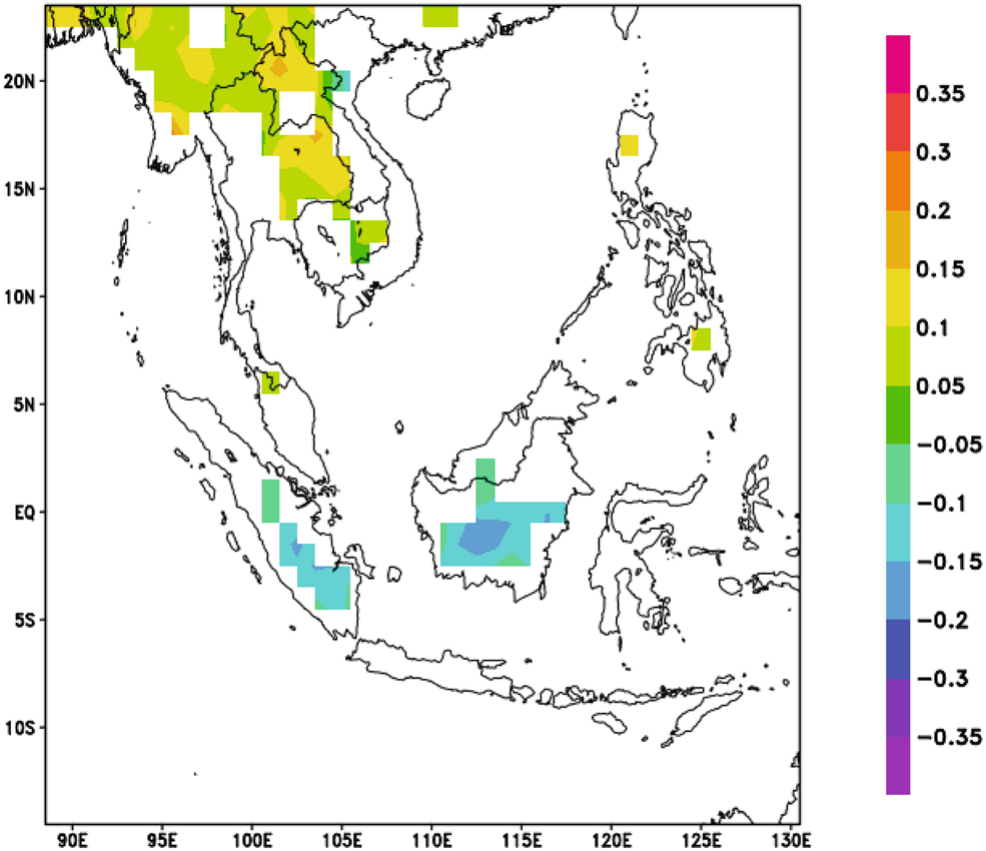
Correlation coefficient values for correlation between AOD and CHIRPS for 2001–2015. Orange indicates significant positive correlation (P-value < 0.05) while blue indicates significant negative correlation (P-value < 0.05). White denotes insignificant correlation over land areas (P-value > 0.05) and missing values over water. The map was generated using Grid Analysis and Display (GrADS) version 2.1.a3 (http://cola.gmu.edu/grads/).

The Pearson correlation analysis was also conducted for the daily precipitation data from CHIRPS and MOIDS AOD data (Fig. 5). From Fig. 5, it can be observed that there are positive correlations between AOD and precipitation over Continental Southeast Asia (Correlation coefficient between +0.1 to +0.3). Some negative correlation coefficients were also observed over some parts of Sumatra and Kalimantan (Correlation coefficient between −0.2 to −0.1). These correlation coefficients are lower than those obtained from the monthly data analysis as the temporal resolution is higher and there will be more “noise” in the data. Thus, the usage of a t-test was essential to ensure that these correlations are significant.

**Figure 6:**
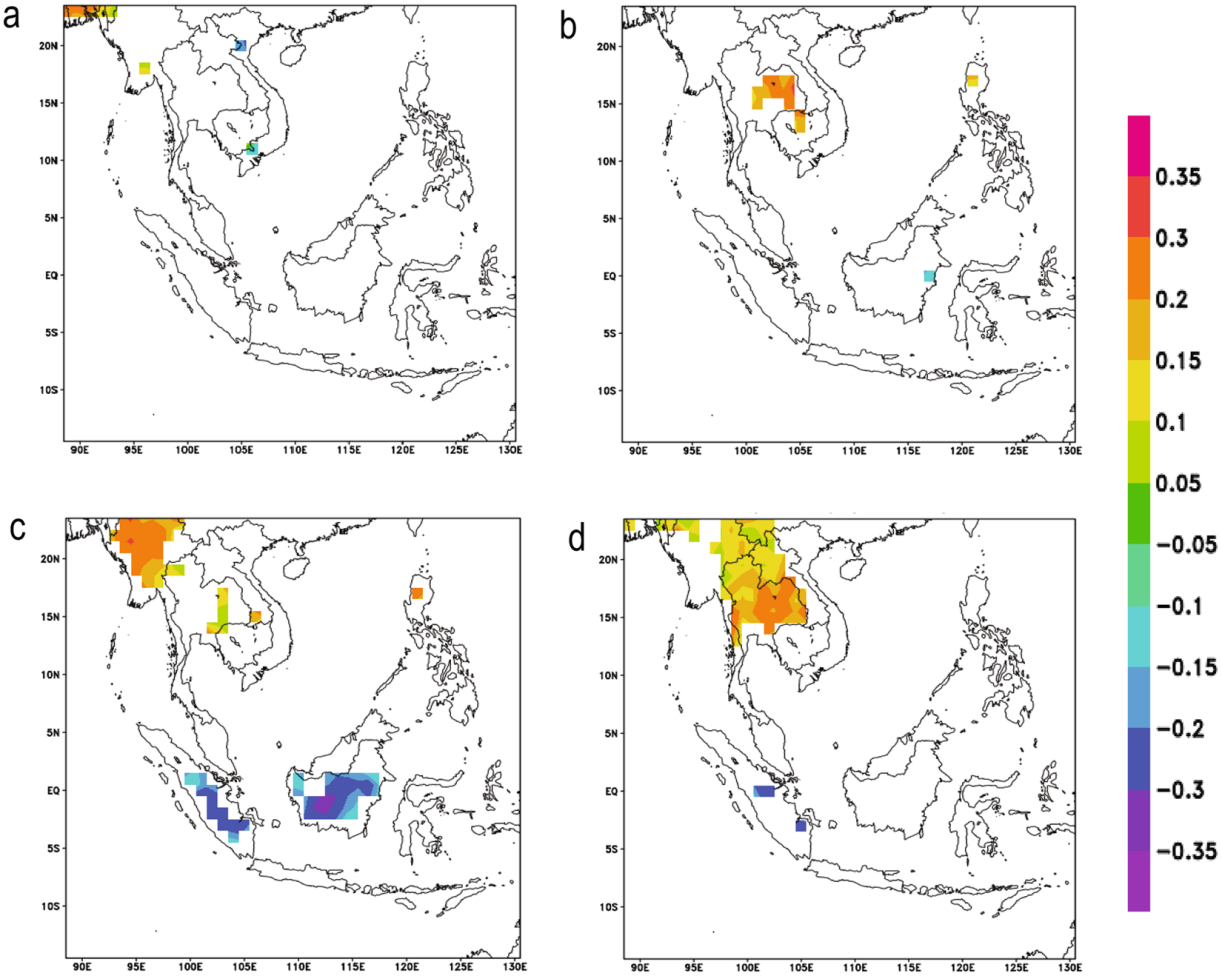
Correlation Coefficient values of correlation between AOD and CHIRPS for 2001–2015 (**a**) March – May, (**b**) June – August, (**c**) September – November and (**d**) December – February. Orange indicates significant positive correlation (P-value < 0.05) while blue indicates significant negative correlation (P-value < 0.05). White denotes insignificant correlation over land areas (P-value > 0.05) and missing values over water. These maps were generated using GrADS version 2.1.a3 (http://cola.gmu.edu/grads/).

Furthermore, when the correlation analysis was repeated by seasons (Fig. 6) for the data from CHIRPS, positive correlations were observed over Continental Southeast Asia in the autumn and winter seasons, while negative correlations were observed over Sumatra and Kalimantan areas only in the autumn season. Sporadic positive correlations were also observed in the spring and summer seasons. These results seem to suggest that the observed relationship between aerosols and precipitation may be spatially different.

While these results of positive correlations are similar to previous studies^19,57^, it is important to note that the correlation alone does not imply causality and it is necessary to account for the covariation between AOD and precipitation with RH. This has been reflected in previous studies which have noted that the variability of the humidity induces perturbations in AOD due to the hygroscopic growth of aerosols^24,27^. In addition to impacting the hygroscopic growth of aerosols and hence altering the aerosols’ physical and optical properties^27^, RH also influences the observed relationship between aerosols and precipitation through cloud processes. With a two-dimensional spectral-bin cloud model, Fan *et al*.^26^ found that the cloud changes from shallow warm to deep convective types when the surface RH increases from 40 to 70%. The resulting convective cloud induces strong precipitation to increase the total amount of precipitation in their simulated convective cloud event occurring on August 24, 2000 in Houston, Texas. Therefore, the favourable high RH conditions for the development of convective cloud could be a reason for the observed positive correlation between precipitation and aerosols.

Furthermore, the negative correlations may not be a reflection of the suppression of precipitation by aerosols as other processes may also result in a negative correlation. Such processes include the wet-scavenging effect which is one of the major sinks for aerosols^22,49,58,59^. Thus, the higher the precipitation, the lower the AOD values. Additionally, during the winter season, both Sumatra and Kalimantan experience dry conditions with minimal precipitation^60^. This leads to widespread forest fires which emit large amounts of aerosols such as black carbon and organic carbon. These dry conditions will also result in a negative correlation between aerosols and precipitation.

### Pearson Correlation Analysis between AOD and RH

**Figure 7:**
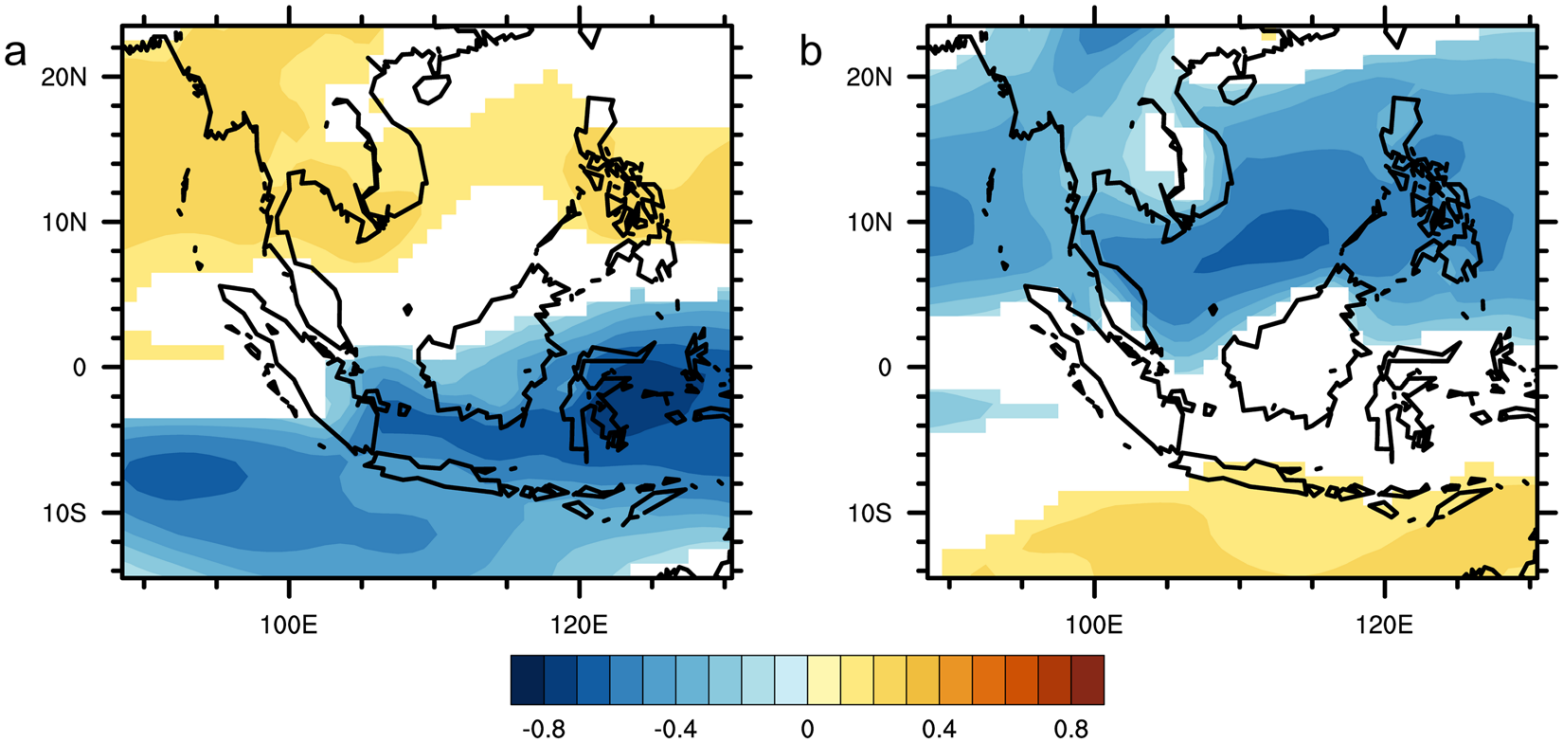
Correlation map between monthly RH and the PCs of AOD for 2001–2015: (**a**) correlation between monthly RH and PC1; (**b**) correlation between monthly RH and PC2. Yellow indicates significant positive correlation (P-value < 0.05) while blue indicates significant negative correlation (P-value < 0.05). White denotes insignificant correlation (P-value > 0.05). These maps were generated using the NCAR Command Language (Version 6.3.0) [Software]. (2015). Boulder, Colorado: UCAR/NCAR/CISL/TDD. 10.5065/D6WD3XH5.

**Figure 8:**
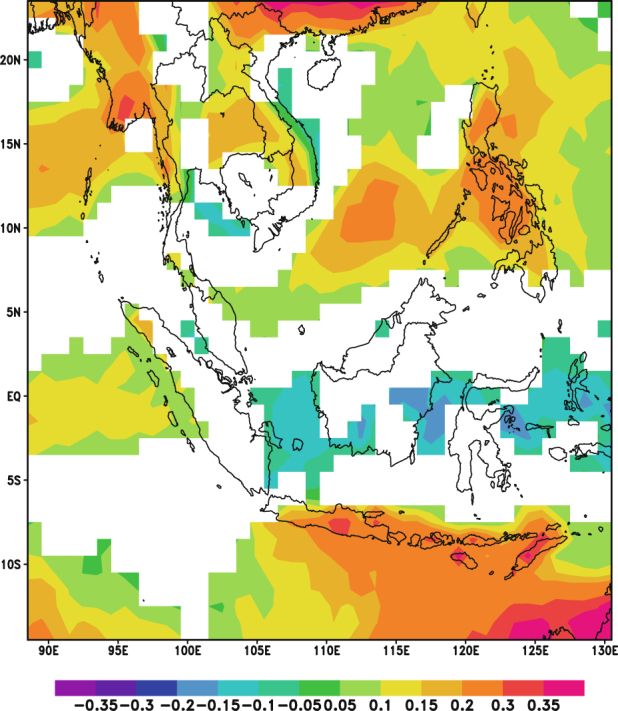
Correlation coefficient values for correlation between AOD and RH for 2001–2015. Orange indicates significant positive correlation (P-value < 0.05) while blue indicates significant negative correlation (P-value < 0.05). White denotes insignificant correlation (P-value > 0.05). The map was generated using Grid Analysis and Display (GrADS) version 2.1.a3 (http://cola.gmu.edu/grads/).

In order to understand the influence of atmospheric humidity on the relationship between aerosols and precipitation, another Pearson correlation analysis was conducted between the AOD and the pressure level averaged RH. From Fig. 7, it can be observed that the correlation map between the PCs of AOD and monthly RH is spatially similar to that of Fig. 4. Furthermore, the negative correlations over the Maritime Continent in Fig. 7a and over the Continental Southeast Asia in Fig. 7b are stronger than that shown in Fig. 4. As previously discussed, these strong negative correlations could be due to dry conditions over the regions which leads to increases in aerosol emissions. Additionally, from Fig. 8, it can be noted that the positive correlations between daily AOD and RH over Continental Southeast Asia are stronger when compared to that in Fig. 5. Thus, it is likely that the relationship between RH and AOD influences the observed relationship between AOD and precipitation.

**Figure 9:**
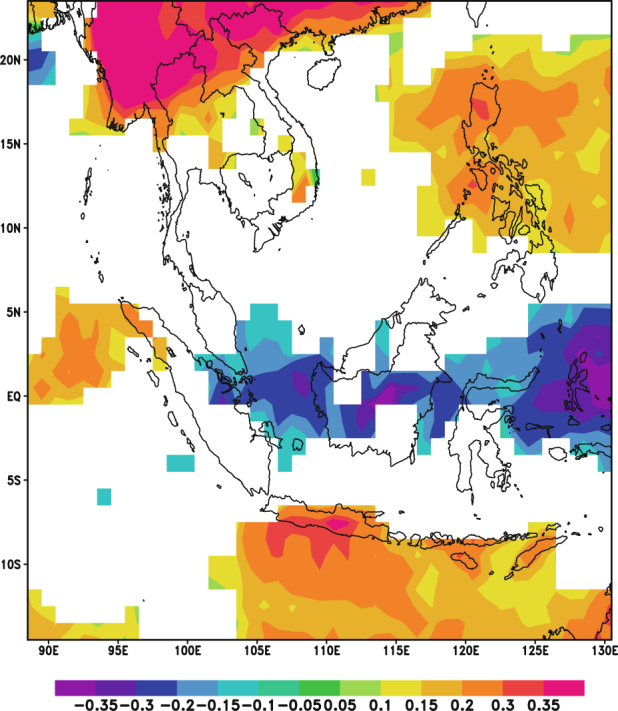
Correlation coefficient values of correlation between AOD and RH for autumn season (September, October and November, 2001–2015). Red indicates significant positive correlation (P-value < 0.05) while purple indicates significant negative correlation (P-value < 0.05). White denotes insignificant correlation over land areas (P-value > 0.05) and missing values over water. These maps were generated using GrADS version 2.1.a3 (http://cola.gmu.edu/grads/).

**Figure 10:**
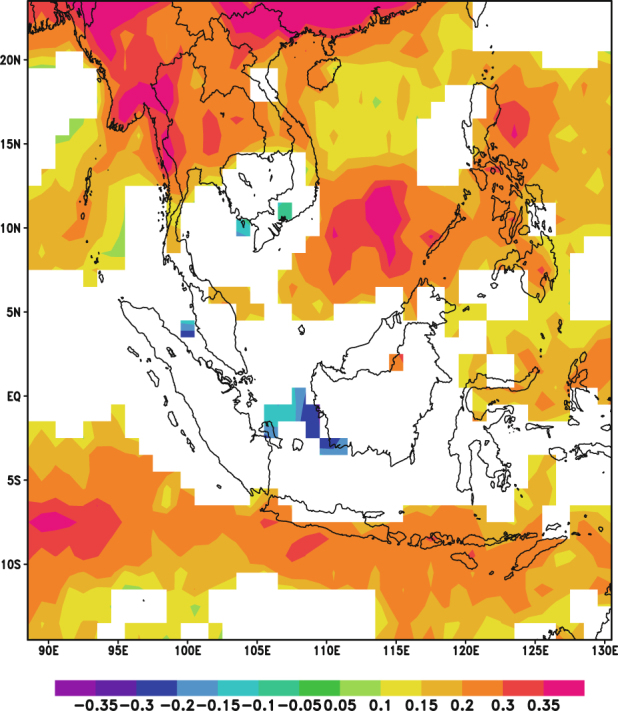
Correlation coefficient values of correlation between AOD and RH for winter season (December, January and February, 2001–2015). Red indicates significant positive correlation (P-value < 0.05) while blue indicates significant negative correlation (P-value < 0.05). White denotes insignificant correlation over land areas (P-value > 0.05) and missing values over water. These maps were generated using GrADS version 2.1.a3 (http://cola.gmu.edu/grads/).

When the analysis was repeated for the pressure level averaged RH over the autumn and winter seasons, stronger positive correlations were also observed over some parts of the Continental Southeast Asia region (Figs 9 and 10). This indicates that observed positive correlations between AOD and precipitation over those areas could be dominated by positive correlations between RH and AOD. Furthermore, over the autumn season, there was also a stronger negative correlation between RH and AOD over some of the areas that previously showed a negative relationship between AOD and precipitation (Fig. 6). Similar to that the analysis for AOD and RH in the monthly scale, the negative correlations could be due to an increase in aerosol emissions during dry conditions.

### Partial Correlation Analysis between AOD and Precipitation

**Figure 11:**
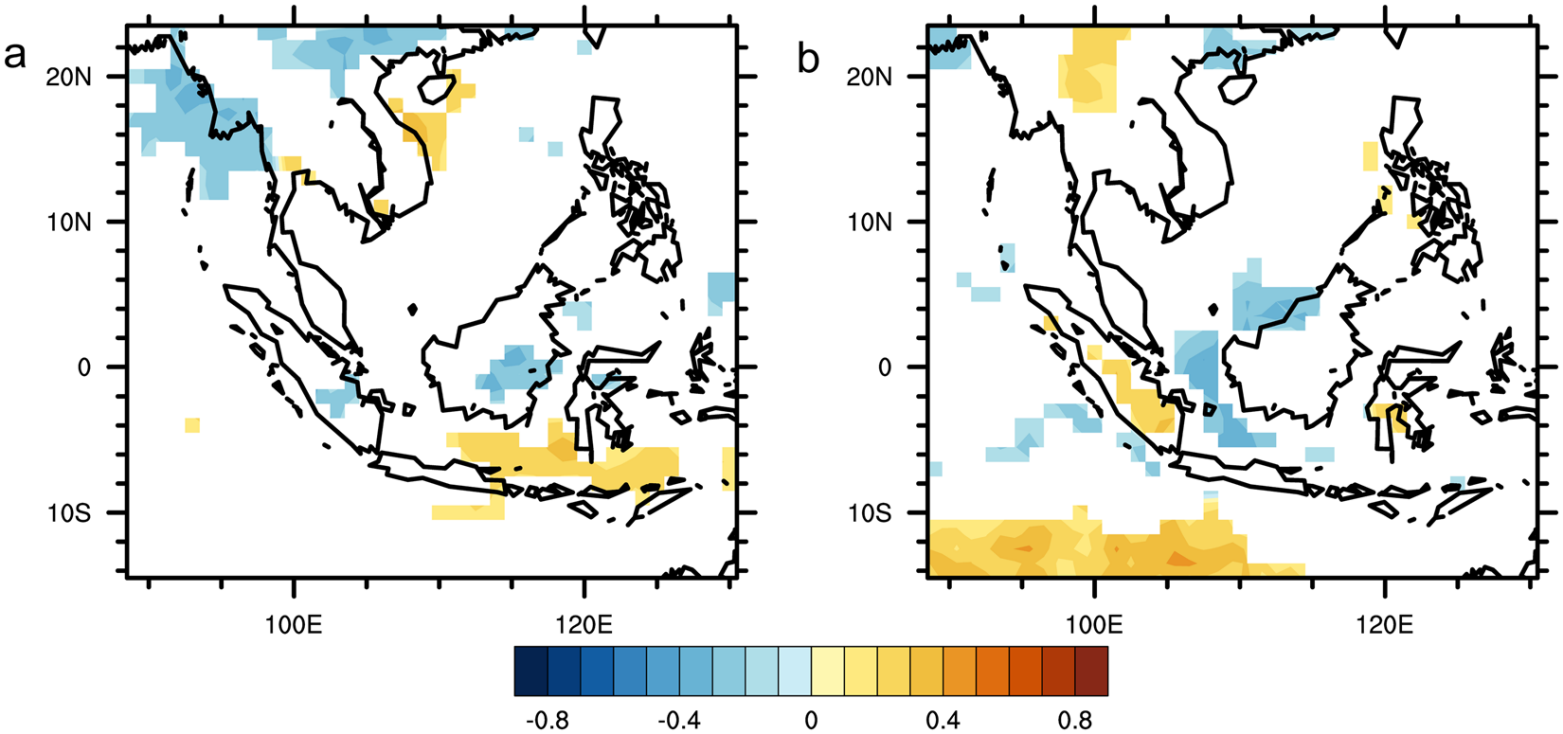
Partial correlation maps between TRMM monthly precipitation and the PCs of AOD for 2001–2015: (**a**) partial correlation map between monthly precipitation and PC1; (**b**) partial correlation map between monthly precipitation and PC2. Yellow indicates significant positive correlation (P-value < 0.05) while blue indicates significant negative correlation (P-value < 0.05). White denotes insignificant correlation (P-value > 0.05). These maps were generated using the NCAR Command Language (Version 6.3.0) [Software]. (2015). Boulder, Colorado: UCAR/NCAR/CISL/TDD. 10.5065/D6WD3XH5.

A partial correlation analysis was conducted to examine the role of RH in the negative correlation between precipitation and the PCs of AOD. The results for the partial correlation analysis on TRMM precipitation are presented in Fig. 11.

Compared against the correlation map shown in Fig. 4a, the partial correlation map (Fig. 11a) shows weaker correlations between monthly precipitation and the PCs of AOD over the Maritime Continent. Similarly, after removing the linear effect of RH on precipitation and AOD, the decrease in the correlation coefficient between these variables within the Continental Southeast Asia region is also seen through the comparison of the partial correlation map shown in Fig. 11b against the correlation map presented in Fig. 4b. The reduction in the strength of the correlation might be explained by the earlier mentioned wet-scavenging effect. The environment with high RH is favorable for precipitation and most aerosols would be removed from the atmosphere through wet scavenging. Comparing with the high RH scenario, the environment with low RH features less precipitation^25^, which set up dry conditions favorable for fire burning thus, retaining aerosols particles in atmosphere^61^. These results indicate that the negative correlations between monthly precipitation and the PCs of AOD in the Maritime Continent and Continental Southeast Asia regions are mainly derived from fluctuations in atmospheric saturation rather than the interactions between aerosols and precipitation.

**Figure 12:**
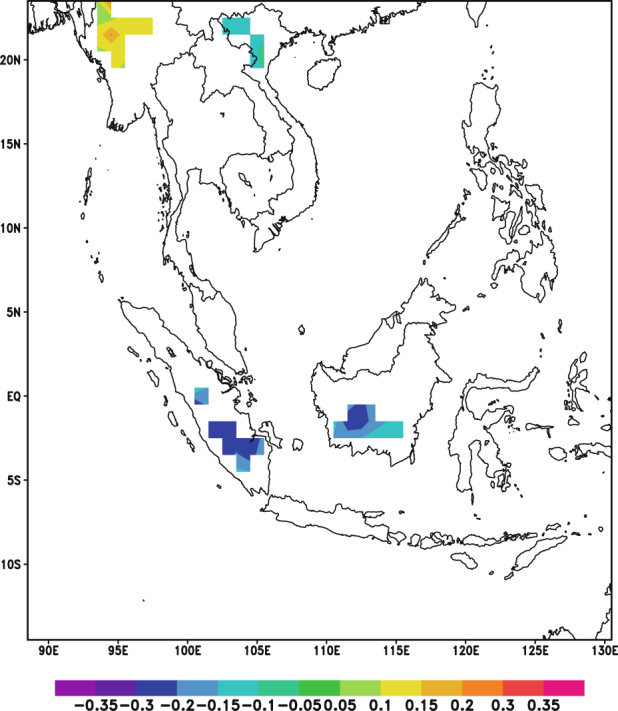
Correlation coefficient values for partial correlation between AOD and CHIRPS for autumn season (September, October and November, 2001–2015). Orange indicates significant positive correlation (P-value < 0.05) while blue indicates significant negative correlation (P-value < 0.05). White denotes insignificant correlation over land areas (P-value > 0.05) and missing values over water. These maps were generated using GrADS version 2.1.a3 (http://cola.gmu.edu/grads/).

A partial correlation analysis during the autumn and winter seasons was also conducted for the CHIRPS dataset. The partial correlation analysis for the autumn season was conducted with pressure level averaged RH (Fig. 12) and the areas which previously showed either positive or negative correlations only showed sporadic correlations. Thus, it can be inferred that most of the observed correlations between AOD and precipitation in the autumn season were due to the influence of RH either through the effect of hygroscopic growth or the increase in aerosols due to the dry conditions.

**Figure 13:**
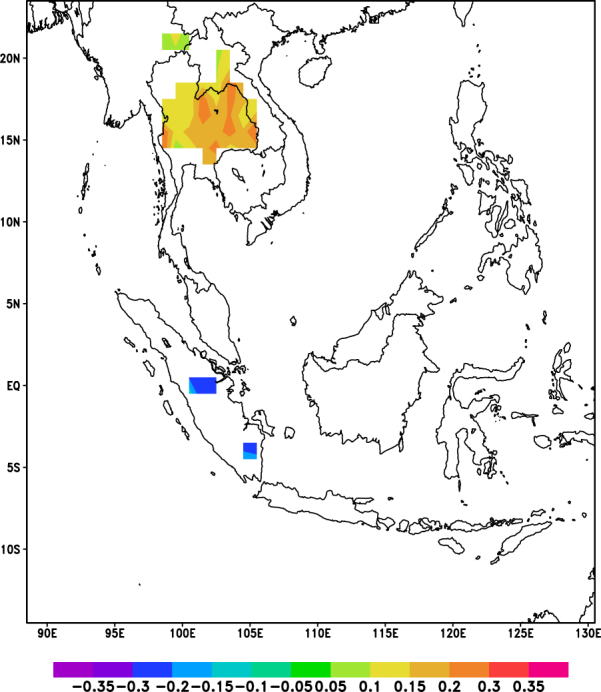
Correlation coefficient values for partial correlation between AOD and CHIRPS for winter season (December, January and February, 2001–2015). Orange indicates significant positive correlation (P-value < 0.05) while blue indicates significant negative correlation (P-value < 0.05). White denotes insignificant correlation over land areas (P-value > 0.05) and missing values over water. These maps were generated using GrADS version 2.1.a3 (http://cola.gmu.edu/grads/).

Based on the similar analysis for the winter season (Fig. 13), it was observed that the region bounded by 14.5°N to 18.5°N and 99.5°E to 104.5°E showed significant positive correlations even after controlling the influence of RH. This suggests that RH may not be the dominating factor influencing the relationship between AOD and precipitation in the winter. It may be likely that these correlations are either due to invigoration of clouds by aerosols^10^, which leads to increase in precipitation over that specific region, or other meteorological conditions.

## Summary and Conclusion

This study has investigated the relationship between AOD and precipitation in Southeast Asia using RH as an influencing factor. The major patterns of variability in AOD were derived by the EOF analysis which highlighted the two action centres in the Maritime Continent and the Continental Southeast Asia. The two action centres contributed the most to variations in AOD over 2001 to 2015. Additionally, the results of the Pearson correlation analysis and the partial correlation analysis were compared to examine the influence of RH on the relationship between AOD and precipitation.

The EOF analysis showed that there are two main modes that account for a significant proportion of the variance in AOD. It is inferred that these modes were attributed to the seasonal occurrence of biomass burning in the Maritime Continent and Continental Southeast Asia. A Pearson correlation analysis between the PCs of AOD and monthly precipitation data from TRMM also showed that the precipitation is strongly correlated with PC1 over the Maritime Continent and with PC2 over Continental Southeast Asia.

Subsequently, a separate Pearson correlation analysis between daily AOD and daily precipitation data from CHIRPS showed that correlations between AOD and precipitation seem to be variable over the region with Continental Southeast Asia mainly showing positive correlations and the Maritime Continent mainly showing negative correlations. Also, similar positive and negative correlations were observed in the autumn and winter seasons when the analysis was conducted season-wise.

The Pearson correlation analyses between AOD and RH for the daily and monthly scales revealed that RH may be the dominating influencing factor in the relationship between AOD and precipitation in the region. When a partial correlation analysis was conducted for the monthly TRMM data, it was noted that most of the previously observed strong negative correlations either decreased or were not significant when RH was used as a controlling variable. This finding indicates that RH seems to be the dominating factor that drives the relationship between AOD and precipitation in the monthly scale over Southeast Asia. The results from the same analysis that was conducted for the daily precipitation data from CHIRPS also showed RH to be the dominating factor during the autumn season and over some Northern regions in the winter season.

Positive correlations were also observed over a central portion of Continental Southeast Asia during the winter season in the partial correlation analysis of CHIRPS data. This leads to the possibility that there is either a presence of a different influencing factor in the winter season or that the aerosols are able to invigorate the clouds during the winter season. Thus, while the study was able to show that not all significant correlations between AOD and precipitation were due to aerosol induced effects, aerosol induced effects could still exist over Continental Southeast Asia, especially during the winter season.

These results suggest that investigations of influencing factors are vital when interpreting precipitation-aerosol relationships from observational data. As this current study has only examined the influence of RH on the relationships of precipitation and AOD, it is recommended that further research be undertaken to understand and identify the presence of other possible influencing factors over the Southeast Asia region.

Additionally, as the usage of observational data was not able to accurately determine the underlying mechanisms for the observed positive correlations between AOD and precipitation in the winter season, it may be possible to conduct numerical simulations at cloud resolving scales to better comprehend and attribute changes in precipitation to aerosol effects.
