# Publisher Correction: Investigating the relationship between Aerosol Optical Depth and Precipitation over Southeast Asia with Relative Humidity as an influencing factor

**DOI:** 10.1038/s41598-018-25179-0

**Published:** 2018-05-10

**Authors:** Daniel Hui Loong Ng, Ruimin Li, Srivatsan V. Raghavan, Shie-Yui Liong

**Affiliations:** 1 Department of Civil and Environmental Engineering, National University of Singapore, Singapore, Singapore; 2 Tropical Marine Science Institute, National University of Singapore, Singapore, Singapore; 3 Center for Environmental Sensing and Modeling, Singapore-MIT Alliance for Research and Technology, Singapore, Singapore; 4 Willis Re Inc., London, UK

Correction to: *Scientific Reports* 10.1038/s41598-017-10858-1, published online 17 October 2017

In the original HTML version of this Article, Daniel Hui Loong Ng was incorrectly listed as the corresponding author. The correct corresponding author for this Article is Ruimin Li. Correspondence and request for materials should be addressed to: li_ruimin@u.nus.edu

This has now been corrected in the HTML version of the paper; the PDF was correct from the time of publication.

